# Unity Assumption in Audiovisual Emotion Perception

**DOI:** 10.3389/fnins.2022.782318

**Published:** 2022-03-04

**Authors:** Ka Lon Sou, Ashley Say, Hong Xu

**Affiliations:** ^1^Psychology, School of Social Sciences, Nanyang Technological University, Singapore, Singapore; ^2^Humanities, Arts and Social Sciences, Singapore University of Technology and Design, Singapore, Singapore

**Keywords:** multisensory integration, semantic emotion perception, unity assumption, autistic traits, Weak Central Coherence Theory

## Abstract

We experience various sensory stimuli every day. How does this integration occur? What are the inherent mechanisms in this integration? The “unity assumption” proposes a perceiver’s belief of unity in individual unisensory information to modulate the degree of multisensory integration. However, this has yet to be verified or quantified in the context of semantic emotion integration. In the present study, we investigate the ability of subjects to judge the intensities and degrees of similarity in faces and voices of two emotions (angry and happy). We found more similar stimulus intensities to be associated with stronger likelihoods of the face and voice being integrated. More interestingly, multisensory integration in emotion perception was observed to follow a Gaussian distribution as a function of the emotion intensity difference between the face and voice—the optimal cut-off at about 2.50 points difference on a 7-point Likert scale. This provides a quantitative estimation of the multisensory integration function in audio-visual semantic emotion perception with regards to stimulus intensity. Moreover, to investigate the variation of multisensory integration across the population, we examined the effects of personality and autistic traits of participants. Here, we found no correlation of autistic traits with unisensory processing in a nonclinical population. Our findings shed light on the current understanding of multisensory integration mechanisms.

## Highlights

- Multisensory integration of emotion intensity follows a Gaussian distribution.
- Angry requires more similar audiovisual intensity to form “Unity” judgment than happy.
- Angry emotion relies more on auditory input, but happy relies more on visual input.

## Introduction

In daily life, we constantly experience stimuli from various sensory modalities in our environment. It has been postulated that these stimuli are processed in modality-specific unisensory systems before integration in the multi-modal process for coherent perception, known as multisensory integration (Meredith and Stein, 1983; Choi et al., 2018). However, increasing evidence has recently suggested this integration to occur even earlier for auditory, visual, and somatosensory input at various subcortical layers and the sensory periphery (Driver and Noesselt, 2008; Bizley et al., 2016; Wang et al., 2017; Gruters et al., 2018). Yet, the extent at which multisensory integration occurs in each area remains highly debated (Rohe and Noppeney, 2016). The strength of such integration also varies according to cue congruency. When stimuli are congruent, the multisensory effect is typically strong. Particularly in spatial integration, percepts tend to be biased toward either auditory or visual information according to their relative reliabilities (Alais and Burr, 2004; Mihalik and Noppeney, 2020). In more complex perception, two mismatched stimuli may even be integrated to create an illusion such as the McGurk effect in which a presented face with the mouth displaying “ga” accompanied with an auditory sound “ba” generates the illusory perception of “da” in more than 75% of adults. However, only about half of children experience such illusory effects, with the other half experiencing auditory dominance in such audiovisual conflicts (McGurk and MacDonald, 1976; Robinson and Sloutsky, 2004). When such unimodal stimuli are weak in intensity or presented with poor signal-to-noise ratio, a strong audiovisual benefit—the principle of “inverse effectiveness” enhances perception of the weak signals through multisensory integration (Meredith and Stein, 1986; Angelaki et al., 2009; Stevenson and James, 2009; van de Rijt Luuk et al., 2019). Taken together, this raises the question: How weak of an intensity is needed to trigger inverse effectiveness in multisensory integration?

The “unity assumption” has proposed a perceiver’s belief of individual unisensory information belonging together to modulate the degree of multisensory integration, particularly in higher-level factors such as speech signals (Chen and Spence, 2017; Vatakis et al., 2018; Uno and Yokosawa, 2021). The closer two pieces of sensory information are spatially, temporally, and semantically, the stronger the unity assumption belief would be, making multisensory integration more likely to occur (Wallace et al., 2004; Wozny and Shams, 2011). This is heavily reliant on causal inference, the implicit perceptual process whereby perceivers determine if signals come from a common, or separate cause (Shams and Beierholm, 2010; Mihalik and Noppeney, 2020). While spatial and temporal limits of the unity assumption have been widely examined (for reviews, see Vroomen and Keetels, 2010; Chen and Vroomen, 2013), the semantical limit has rarely been investigated. In this study, we aimed to understand how semantically similar in emotion intensity for the face and voice to meet unity assumption, and how meeting this assumption would affect multisensory integration strategies.

While low-level audiovisual perception may rely only on spatial and temporal congruence, an additional feature—semantic congruence—plays an increasingly important role as audiovisual stimuli get more complex, e.g., in speech, animal videos or emotion expression perception (de Gelder and Vroomen, 2000; Chen and Spence, 2017). Typically, it has been found that when semantic categories of the visual and auditory stimuli are congruent, the categorization accuracy and reaction time would be facilitated (de Gelder and Vroomen, 2000; Collignon et al., 2008; Koppen et al., 2008; Chen and Spence, 2010). However, unlike spatial and temporal congruence, semantic congruence is usually categorical (i.e., Congruent vs. Incongruent), and is rarely quantified in the literature. The lack of quantification results in the inability to examine the exact criteria for semantic aspects of the unity assumption. In fact, under its strict definition, it can be argued that the semantic congruence effect is irrelevant to the unity assumption as it is rare to directly ask if subjects perceive audiovisual stimuli to correspond to the same object/event (Chen and Spence, 2017). This raises the question of the role semantic congruence plays in the integration of audiovisual stimuli.

This study, therefore, attempts to address the above questions by quantifying semantic congruence in the context of audiovisual emotion perception. Here, semantic congruence was measured as the degree of emotional intensity similarity in audiovisual stimuli (a face and voice), and their respective sources—whether from the same person or not, as perceived by subjects. This enabled us to then identify a critical face-voice emotional intensity difference for meeting the unity assumption. Concurrently, we also explored any difference in the strategies used in multisensory emotion perception when the unity assumption was met, as compared to when it was not. As previous studies have demonstrated multisensory emotion perception to be visually dominant when the intensities of audiovisual emotions were incongruent (Collignon et al., 2008), we hypothesize that facial information contributes more toward multisensory emotion perception than vocal information when the unity assumption is not met, but expect equal contributions when it is.

The ability for multisensory integration is known to vary across the population (McGurk and MacDonald, 1976; Robinson and Sloutsky, 2004; Laurienti et al., 2006; Charbonneau et al., 2013). As predicted by the Weak Central Coherence Theory (Happé and Frith, 2006), Autism Spectrum Disorder (ASD) and autistic traits have been associated with disrupted holistic perception (Behrmann et al., 2006; Nakahachi et al., 2008; Luo et al., 2017). In multisensory perception of emotion, it was found that reaction time facilitation of autistic individuals was less than that of typically developed individuals (Charbonneau et al., 2013). This atypical integration of multisensory input might explain the impaired social cognitive abilities in ASD (Kawakami et al., 2020a). Though results have previously shown autistic individuals to be unable to successfully integrate the two separate sensory stimuli, the underlying mechanisms for this impairment in multisensory emotion perception still remain unclear. One recent theory attempting to address this is the Bayesian model of autistic perception (Pellicano and Burr, 2012; Sevgi et al., 2020). The theory postulates that the social/non-social perceptual deficits in autism result from a deviation in the construction of top-down influences, i.e., priors, and/or the reduction in use of such priors. One of these priors in multisensory integration is the unity assumption, where the observer perceives two different sensory signals to be coming from the same source (Körding et al., 2007; Shams and Beierholm, 2010; Chen and Spence, 2017). In support of this, one recent study showed that individuals with higher autistic traits tended to have a narrower temporal window for audiovisual integration than those with lower autistic traits during low-level multisensory perception (Kawakami et al., 2020b). In a similar thread, could autistic individuals also have stricter criterion for the semantic aspect of the unity assumption? In this case, the reduced multisensory benefit for emotion perception in autistic individuals may be explained as a reduced ability to perceive emotionally charged facial and auditory cues to be from the same person. However, the opposite has also been reported (Poole et al., 2017; Kawakami et al., 2020a), suggesting that this may not be a universal phenomenon.

Another prediction of the Bayesian model of autistic perception is that autistic individuals tend not to use the prior during multisensory perception (Pellicano and Burr, 2012; Sevgi et al., 2020). Accordingly, autistic traits should therefore attenuate the strategic change in audiovisual integration when the unity assumption is either met, or not. The autistic deviation in audiovisual emotion perception may be expected to occur only when the unity assumption is met. To test this hypothesis, we may lend theoretical understanding toward the autistic influence in multisensory emotion perception: If the change occurs when the “unity assumption” is met, it indicates autistic influences in multisensory emotion perception to be partially due to a deviation in top-down influences; though if it occurs regardless of whether the unity assumption is met, it might instead point toward the autistic influence occurring at the stage independent from top-down influences, i.e., either before integration or at a unimodal stage.

Therefore, we aimed to address two questions in this study: (1) How similar would the emotional intensities of the face and voice be to meet the “unity assumption,” and how would this then affect an individual’s audiovisual integration strategy? This question was addressed directly by asking if subjects perceived audiovisual stimuli to be similar in emotional intensity such that the face and voice were possibly coming from the same person. By comparing the differences in emotional intensities of the face and voice of subjects’ judgments, we identified the point at which the unity assumption came into effect. Next, by modeling subjects’ responses, we could obtain their reliance on individual visual or auditory information for comparison across conditions. (2) How do autistic traits affect the criteria of the “unity assumption” and audiovisual integration strategy? To answer this question, we included measures of autistic traits, alexithymia, depression, anxiety, and stress to examine their effects and control for the influence of such common comorbidities of autism (Mazzone et al., 2012; Bird and Cook, 2013; Poquérusse et al., 2018).

## Materials and Methods

### Participants

Forty-eight subjects (mean age = 21.69, *SD* = 1.85; 36 females) consented to and participated in the experiment at the lab. All participants met the inclusion criteria of being University students and having normal, or corrected-to-normal vision, with no diagnosed clinical or neurological impairments. Sample size was determined based on previous studies (Brandwein et al., 2015; West et al., 2018). This study was approved by the Institutional Review Board (IRB) at Nanyang Technological University, Singapore, by the Code of Ethics of the World Medical Association (Declaration of Helsinki) for experiments involving human subjects.

### Stimulus

Facial stimuli were obtained from the Karolinska Directed Emotional Faces—Dynamic (KDEF-dyn) database (Lundqvist et al., 1998; Calvo et al., 2018). The dynamic faces were 30-frame videos, each a frame-by-frame morph first from a neutral (frame 1) to emotional (frame 30) face of the same actor. Videos of angry and happy faces of 4 actors (2 females; face ID F02, F09, M06, and M11) were selected based on high recognition rates (> 90%) as observed in a previous validation study (Calvo et al., 2018). From the original videos, we further derived five video types, each representing a different condition: (1) Angry Strong: 30-frame video from neutral to 100% angry (30th frame of original angry video); (2) Angry Weak: 15-frame video from neutral to 50% angry (15th frame of original angry video); (3) Happy Strong: 30-frame video from neutral to 100% happy (30th frame of original happy video); (4) Happy Weak: 15-frame video from neutral to 50% happy (15th frame of original happy video); (5) Neutral: 1st frame of the videos (0% angry/happy). In the 30- and 15-frame videos, each frame lasted for 40 and 79 ms, respectively, with the last frame presented for an additional 500 ms to mitigate the effects of an abrupt end to the video. Together, the duration of each video was 1.69 s. Faces in the neutral condition were also presented for 1.69 s. All frames were converted to grayscale and matched in luminance using MATLAB R2018b (Mathworks, MA, United States) using the toolbox SHINE (Willenbockel et al., 2010). Faces were consistently presented at 6.5 inches × 5 inches. In total, there were 20 facial stimuli videos (5 emotional conditions × 2 genders × 2 actors per gender).

Voice stimuli were obtained from the dataset Ryerson Audio-Visual Database of Emotional Speech and Song (RAVDESS) (Livingstone and Russo, 2018). Strong Angry voice, Weak Angry voice, Strong Happy voice, Weak Happy voice, and Neutral voice of 4 actors (2 females; voice IDs are F08, F14, M03, and M15) were similarly selected based on the high recognition rates observed in a previous validation study (≥ 80%, except one weak happy voice clip with 40% accuracy and one strong happy voice clip with 70% accuracy) (Livingstone and Russo, 2018). Each clip depicted an actor speaking an emotionally neutral sentence (“Kids are talking by the door,” “Dogs are sitting by the door”) either in an emotional or neutral voice. Voice volume intensities were standardized using Praat 6.1.12 (Boersma, 2001). The average duration of voices was 1.69 s (*SD* = 0.31 s), to match the duration of face stimuli. In total, there were 20 voice stimuli clips (5 emotional conditions × 2 genders × 2 actors per gender). The voices were presented from the computer (iMac) speaker, the volume adjusted such that the voices could be clearly heard.

The multisensory stimuli were then generated by combining the face and voice stimuli. Only stimuli of the same gender and emotional category (except neutral stimuli which were constructed of both angry and happy) were combined. As a result, there were 144 multisensory stimuli (2 emotions (Angry and Happy) × 9 intensity combinations (3^2^ intensities: Neutral, Weak, and Strong) × 4 actor combinations (2^2^ actors) × 2 genders). The duration of each frame of face stimulus was calculated as: (Duration of the Voice Stimulus—0.5 s)/Total Number of Frames in the Face Stimulus. For example, if a 30-frame face stimulus was combined with a 1.2 s voice stimulus, the duration of each frame would be (1.2–0.5 s)/30 frames = 23 ms. Again, the last frame was presented for an additional 500 ms to mitigate the effects of an abrupt end to the video. For Neutral face stimuli, the face was presented for the same duration as the voice stimulus.

### Apparatus

All visual and auditory stimuli were presented on a 27-inch iMac with a refresh rate of 60 Hz and spatial resolution of 1,920 × 1,080 pixels. The behavioral tasks were conducted in MATLAB R2018b (Mathworks, MA, United States) with the Psychophysics Toolbox extensions (Brainard, 1997; Pelli, 1997). The questionnaires were presented in Google forms. The experiment was conducted in a dim-lit room of a quiet psychophysics lab.

### Procedure

Upon reading the study information and indicating participatory consent, subjects then proceeded to first complete either the questionnaires or behavioral tasks in an order randomized across subjects. Three questionnaires—the Autism Spectrum Quotient (AQ) (Baron-Cohen et al., 2001), Toronto Alexithymia Scale (TAS-20) (Bagby et al., 1994) and Depression, Anxiety, Stress Scale (DASS-21) (Lovibond and Lovibond, 1995) were completed in a randomized, consecutive order.

In the behavioral task session, subjects first familiarized themselves with the stimulus validation task through a practice block. This block also served as time for volume calibration to ensure clear presentation of the voice stimuli. After, subjects then completed the stimulus validation block in which they identified the emotions of the face stimuli or the voice stimuli from the choices “Angry,” “Happy,” “Neutral,” and “None of the above.”

**FIGURE 1:**
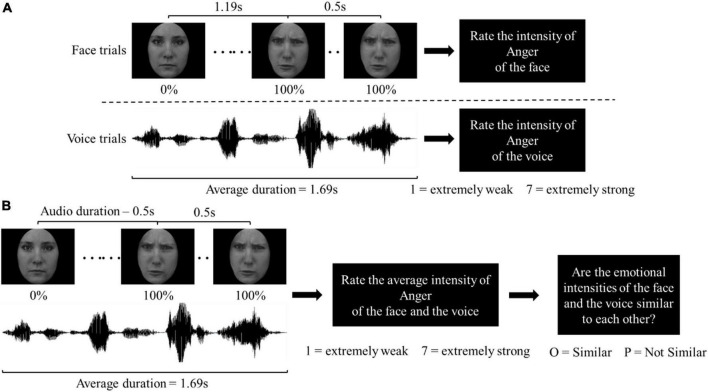
Trial sequence in **(A)** the unisensory block; **(B)** the multisensory block. For demonstration purposes, a trial sequence with Strong Angry stimuli is illustrated. In the Weak condition, the duration of each frame was extended accordingly so the face sequence ended at 50% and stayed for 500 ms. In the Neutral condition, the presented face was always 0% Angry/Happy throughout the face sequence.

Next, there was a second practice block for subjects to then familiarize themselves with the Unisensory and Multisensory tasks. The Unisensory and Multisensory blocks were completed in a randomized order. In the Unisensory block (Figure 1A), either a face or voice stimulus was presented on screen, or from the audio speaker only for an average of 1.69 s. After each presentation, subjects rated the emotional intensity of the stimulus on a 7-point Likert scale. For the Angry or Happy stimuli, each was presented twice throughout the block, subjects being asked to rate the intensity of anger or happiness, respectively. For Neutral stimuli, each was presented four times throughout the block, with subjects asked to rate the intensity of anger in half of the trials, and the intensity of happiness in the other half. As a result, there were a total of 96 trials in the Unisensory block, of 2 modalities (Face and Voice) × 2 emotions (Angry and Happy) × 3 intensities (Neutral, Weak, and Strong) × 2 actors × 2 genders × 2 repetitions.

In the Multisensory block (Figure 1B), a multisensory stimulus was also presented on screen and from the speaker for an average of 1.69 s. After presentation, subjects were asked to rate the emotional intensity of the stimulus on a 7-point Likert scale. Each of the Angry and Happy stimuli was presented once throughout the block. Subjects were asked only to rate the intensity of anger for Angry stimuli, and the intensity of happiness in Happy stimuli. For Neutral stimuli, each stimulus was shown twice throughout the block, subjects asked to rate the intensity of anger in half, and the intensity of happiness in the other half of the trials. After the intensity ratings, the subjects were also asked to judge: “Are the emotional intensities of the face and the voice similar to each other?” Subjects pressed “O” on a keyboard to indicate if intensities were Similar and “P” if they were Not Similar. “Emotional intensities are similar” was defined as “it is possible that the face and voice are coming from the same person” at the beginning of the block. In total, there were 144 trials in the Multisensory block: 2 emotions (Angry and Happy) × 9 intensity combinations (3^2^ intensities: Neutral, Weak, and Strong) × 4 actor combinations (2^2^ actors) × 2 genders.

### Statistical Analysis

In the Multisensory block, trials were categorized by two factors: (1) Similarity—Similar vs. Not Similar trials in which the face-voice pair was judged as “similar” or “not similar” in emotional intensity; and (2) Emotion—Angry vs. Happy trials in which the subjects were asked to rate the intensity of anger and happiness, respectively. One of our aims was to identify how emotionally different the face and voice had to be for perceivers to believe the two were from/not from the same person (i.e., meeting/not meeting the “unity assumption”). To achieve this, we conducted the Receiver Operating Characteristic (ROC) analysis (Greiner et al., 2000) on audiovisual emotional intensity differences in order to classify the two states for each subject. Audiovisual emotional intensity differences were calculated by subtracting the average intensity rating for voices in all emotions from the average intensity rating of faces in all emotion conditions. These were calculated based on ratings in the validation (unimodal) trials only. The optimal cut-off was identified by maximizing the Youden index (J = Sensitivity + Specificity − 1) (Hilden, 1991). If more than one cut-off showed the same Youden index, we chose the one with minimum differences in sensitivity and specificity. A one-way (Emotion) within-subject Analysis of Covariance (ANCOVA) was conducted to investigate if the cut-off value differed across emotions. Normalized AQ, TAS-20, Depression, Stress and Anxiety scores were included in the analysis as covariates.

Another aim was to identify the individual contributions of the face and voice during multisensory emotion perception and to examine if these contributions differed across conditions. To achieve this aim, the intensity rating responses of each subject were fitted into the following linear regression model:


$$Intensity_{M}=\beta_{V}Intensity_{V}+\beta_{A}Intensity_{A}+c$$

Where *Intensity*_*M*_, *Intensity*_*V*_, and *Intensity*_*A*_ represent the emotional intensity ratings of the multisensory stimulus (from the Multisensory block), the face (from the visual trials of the Unisensory block), and the voice (from the auditory trials of the Unisensory block) respectively. Coefficients β_*V*_ and β_*A*_ represent weights of the emotional intensities of the face and voice, respectively, in predicting the emotional intensity of the multisensory stimulus, and coefficient *c* representing the remaining factors not captured by face or voice.

The sensory reliance score was calculated by subtracting β_*A*_ from β_*V*_ such that the more positive the sensory reliance was, the more a subject was relying on visual input during multisensory perception, whereas the more negative the sensory reliance score was, the heavier the subject’s reliance on auditory input was. When the sensory reliance score was equal to zero, the visual input and the auditory input were equally weighted in multisensory perception. After, a 2 (Similarity) × 2 (Emotion) within-subjects ANCOVA was conducted to compare the sensory reliance scores across conditions. The unisensory degree score was calculated by taking the absolute value of the sensory reliance score. The higher the unisensory degree score was, the heavier the subject’s reliance on unisensory input during multisensory perception, whereas the lower the unisensory degree score, the heavier the subject’s reliance on both sensory input during multisensory perception. Furthermore, 2 (Similarity) × 2 (Emotion) within-subjects ANCOVA was conducted to compare the unisensory degree scores across conditions. In both ANCOVAs, covariates of normalized AQ, TAS-20, Depression, Anxiety, and Stress scores were included.

Statistical analysis was conducted using SPSS Statistics 25 (IBM, NY, United States) and Matlab R2018b (Mathworks, MA, United States).

## Results

### Behavioral Response Accuracy and Unisensory Emotional Intensity Rating

**FIGURE 2:**
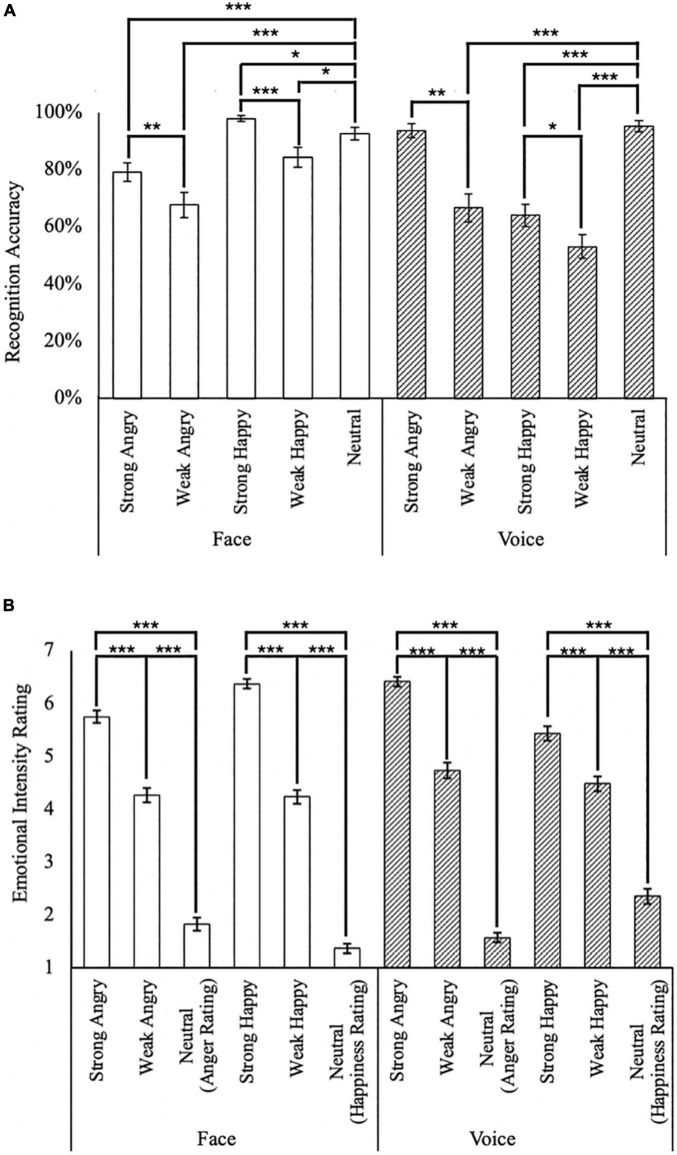
Summary of behavioral responses. **(A)** Emotion recognition accuracy for face (white bars) and voice (hatched bars); and **(B)** unisensory emotional intensity rating. *p < 0.05, **p < 0.01, ***p < 0.001. The error bars indicate standard errors of mean (SEM).

Unisensory stimuli presented in the validation block generally achieved high recognition rates (> 74%; Figure 2A), with the exception of Weak Angry faces (*M* = 67.7%, *SD* = 30.5%), as well as Weak Angry (*M* = 66.7%, *SD* = 33.6%), Strong Happy (*M* = 64.1%, *SD* = 26.8%), and Weak Happy (*M* = 53.1%, *SD* = 28.5%) voices. Emotions in facial stimuli appeared to be better recognized than voice stimuli [*t*(47) = 3.96, *p* < 0.001], with high average recognition rates of both faces (*M* = 84.4%, *SD* = 11.7%) and voices (*M* = 74.6%, *SD* = 13.3%).

In facial stimuli, stronger happy (*M* = 97.9%, *SD* = 7%) intensities were better recognized compared to weaker happy intensities [*M* = 84.4%, *SD* = 24.5%; *t*(47) = 4.42, *p* < 0.001]. Similarly in conditions for the angry emotion, stronger angry (*M* = 79.2%, *SD* = 22.1%) intensities were better recognized than weaker angry displays [*M* = 67.7%, *SD* = 30.5%; *t*(47) = 3.02, *p* < 0.001]. Interestingly, neutral faces (*M* = 92.7%, *SD* = 15.4%) were better recognized than both Strong and Weak Angry faces [*t*(47) = 3.44, *p* = 0.001, and *t*(47) = 4.53, *p* < 0.001, respectively], but significantly less accurately than Strong Happy faces [*t*(47) = 2.48, *p* = 0.02].

In contrast, auditory stimuli of stronger intensities were better recognized than weak intensity conditions for both angry [*t*(47) = 5.92, *p* < 0.001, with strong angry voices: *M* = 93.8%, *SD* = 16.7% and weak angry voices: *M* = 66.7%, *SD* = 33.6%] and happy emotions [*t*(47) = 2.17, *p* = 0.035, with strong happy voices: *M* = 64.1%, *SD* = 26.8%, and weak happy voices: *M* = 53.1%, *SD* = 28.5%]. Different from faces, the neutral voice (*M* = 95.3%, *SD* = 13.3%) was better recognized than weak angry voices [*t*(47) = 6.51, *p* < 0.001] but not strong angry voices [*t*(47) = 0.77, *p* = 0.44], as well as better than both strong and weak happy voices [*t(*47) = 7.25, *p* < 0.001, and *t*(47) = 8.94, *p* < 0.001, respectively].

In the intensity rating task (Figure 2B), as expected, strong emotion conditions were rated as of higher intensity than weak [*t*(47) = 22.5, *p* < 0.001, with Strong: *M* = 5.99, *SD* = 0.62, and Weak: *M* = 4.43, *SD* = 0.79] and neutral conditions [*t*(47) = 37.1, *p* < 0.001, with Neutral: *M* = 1.78, *SD* = 0.63]. Interestingly, angry voices were rated as of higher intensity than angry faces (*t*(47) = 4.46, *p* < 0.001, with Face: *M* = 3.94, *SD* = 0.70, and Voice: *M* = 4.24, *SD* = 0.60). However, this difference between face and voice was not observed in the happy emotion [*t*(47) = 1.15, *p* = 0.26, with Face: *M* = 3.99, *SD* = 0.54, and Voice: *M* = 4.10, *SD* = 0.81].

### Difference in Emotional Intensities Between Face and Voice to Meet the “Unity Assumption”

**FIGURE 3:**
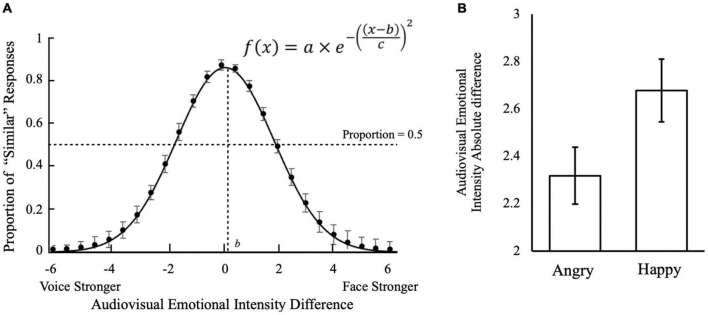
Audiovisual emotion intensity differences in multisensory integration. **(A)** Effect of audiovisual emotion intensity difference on judgment that audiovisual stimuli were similar in emotional intensity. Positive audiovisual emotional intensity differences indicate the emotional intensity of faces to be stronger than voice; negative differences indicate voices to be stronger than faces. The estimated parameters for the Gaussian distribution: *a* = 0.86, 95% CI = [0.73, 1.0]; *b* = 0.21, 95% CI = [0.1, 0.23]; *c* = 0.88, 95% CI = [0.72, 1.04]. **(B)** Face-voice emotional intensity absolute difference cut-off for differentiating Similar trials and Not Similar trials by emotion conditions. Optimal cut-offs were obtained from each participant using ROC analysis by maximizing the Youden index (*J* = Sensitivity + Specificity − 1). The error bars indicate standard errors of mean (SEM) in **(A,B)**.

To understand when subjects judged the face and voice to be of similar emotion intensity, we further analyzed the audiovisual emotional intensity differences and their responses of “Similar” judgment (Figure 3A). Subjects were most likely to judge stimuli to be of “Similar” intensities when the intensity difference was +0.5 (Face stronger; *M* = 85.9%, *SD* = 12.4%), 0 (*M* = 85.1%, *SD* = 16.5%), and −0.5 (Voice stronger; *M* = 83.6%, *SD* = 18.2%). When the audiovisual emotional intensity difference increased to approximately ± 2, the likelihood of making “Similar” judgments dropped below 50%. Fitting the subjects’ responses into a Gaussian curve using the Curve Fitting toolbox in Matlab R2018b (Mathworks, MA, United States), we found that the center of the curve was significantly biased toward the face compared to the voice [*b* = +0.21, 95% *CI* = (0.10, 0.32)]. This suggests that when faces were slightly emotionally stronger than the voice, that subjects would still judge them to be of “Similar” emotion intensities.

We then conducted the ROC analysis to investigate subjects’ criteria for judging audiovisual stimuli to be of a similar emotion. The average cut-off intensity for differentiating “Similar” trials from “Not Similar” trials was 2.50 (*SD* = 0.71). The result suggesting that when the emotional intensity difference between the face and voice was below 2.50 (on a 7-point Likert scale), subjects judged them to be of similar emotion intensity and perceived the face and voice to be from the same person, as defined at the beginning of the experimental block. Thus, the unity assumption would be met when the intensity differences between face and voice was below 2.50 on the 7-point Likert scale.

A one-way analysis of covariance (ANCOVA) on intensity ratings with the main effect of Emotion and covariates AQ, TAS, Depression, Anxiety and Stress revealed a main effect of Emotion [*F*(1, 42) = 5.73, *p* = 0.021, partial η^2^ = 0.12], but no effects of any covariates (*p’*s ≥ 0.25). Further analysis revealed that the optimal cut-off difference (in absolute value) for face-voice emotional intensity to differentiate Similar trials from Not Similar trials was 2.32 (*SD* = 0.83), and 2.68 (*SD* = 0.92) points on the 7-point Likert scale for Angry and Happy emotions, respectively (Figure 3B). It thus suggests that the Angry emotion requires a higher similar audiovisual intensity than the Happy emotion to form a “Unity” judgment.

### Reliance on Face or Voice in Multisensory Integration

**FIGURE 4:**
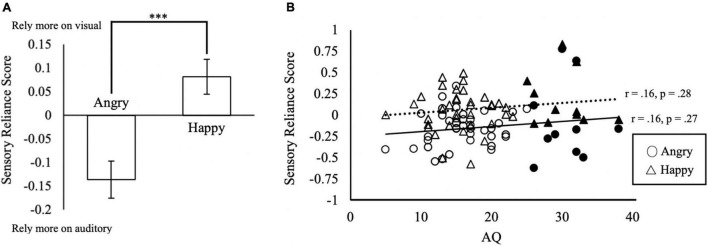
Sensory reliance in multisensory integration. **(A)** Sensory reliance score by emotion conditions. Positive values in sensory reliance score indicate a higher reliance on visual input, negative values indicating a higher reliance on auditory input during multisensory emotion perception. ^***^p < 0.001. The error bars indicate standard errors of mean (SEM). **(B)** Relationship between AQ and sensory reliance score. Solid triangles and circles indicate the responses from the subjects with AQ ≥ 25.

Do participants rely on the face or voice for their judgment? We calculated the sensory reliance score as the difference between auditory and visual coefficients in the linear regression model by β_*V*_−β_*A*_. The 2 (Similarity) × 2 (Emotion) within-subjects ANCOVA (AQ, TAS-20, Depression, Anxiety and Stress as the covariates) on the sensory reliance score showed a significant main effect of *Emotion* [*F*(1, 42) = 46.13, *p* < 0.001, partial η^2^ = 0.52]. The sensory reliance score of the Angry condition (*M* = −0.14, *SD* = 0.31) was significantly different from that of the Happy condition [*M* = 0.08, *SD* = 0.26; *t*(47) = 7.04, *p* < 0.001; Figure 4A]. This suggests that the perception of angry emotion tends to rely more on auditory information, whereas happy emotion relies more on visual information. A significant main effect of Similarity was also observed [*F*(1, 42) = 10.17, *p* = 0.003, partial η^2^ = 0.19]. Sensory reliance scores in the Similar condition (*M* = 0.05, *SD* = 0.22) were significantly higher than that of the Non Similar condition [*M* = −0.10, *SD* = 0.32; *t*(47) = 3.18, *p* < 0.01]. Furthermore, we also found a significant interaction effect of *Emotion × Similarity × Anxiety* [*F*(1, 42) = 6.85, *p* = 0.01, partial η^2^ = 0.14). No other effects related to AQ, TAS-20, Depression, Anxiety, and Stress were found to be significant (*p’*s ≥ 0.09).

### Autistic Traits Do Not Correlate With Sensory Reliance

To investigate whether autistic traits (AQ) were associated with sensory reliance, we correlated AQ with sensory reliance scores using Pearson’s correlations (Figure 4B). Surprisingly, we found no significant correlations of AQ scores with sensory reliance in Happy or Angry conditions (Figure 4B). Overall, the sensory reliance scores of Happy condition are more positive than those of Angry condition. As positive reliance scores indicate reliance on visual signals, this may suggest that the Happy emotion condition elicited heavier reliance on visual signals while the Angry emotion relied more on auditory signals, consistent with findings from the above ANCOVA.

### Testing the Reliability-Weighted Multisensory Integration Model in Similar Trials

When the emotion intensities of both sensory modalities were similar (Similar condition), which sensory modality did the subjects rely on to make their judgments? The reliability-weighted model (Ernst and Banks, 2002; Alais and Burr, 2004) may provide an answer to this. The reliability-weighted model predicts that the weights of sensory information during multisensory integration depend on the relative reliability of sensory information. Therefore, if vision is more situationally reliable than auditory information, visual information will be weighted heavier during multisensory integration; otherwise, auditory information will be weighted heavier.

**FIGURE 5:**
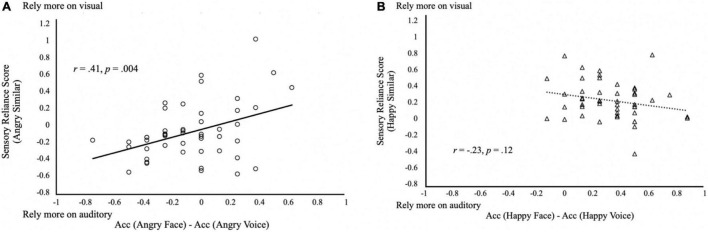
Reliability-weighted multisensory integration model. Relationship between the difference in the audiovisual difference in emotion recognition accuracy and the sensory reliance scores in the Similar trials with **(A)** angry stimuli; and **(B)** happy stimuli. Positive values in sensory reliance score indicate a higher reliance on visual input, and negative values indicate a higher reliance on auditory input during multisensory emotion perception.

To test if subjects’ responses in the Similar trials fitted with the reliability-weighted model, we correlated the subjects’ sensory reliance scores with their audiovisual difference in emotion recognition accuracy (recognition accuracy in face—recognition accuracy in voice). Surprisingly, results showed that the two were not significantly correlated with each other (*r* = 0.13, *p* = 0.41). However, to further explore whether there was an effect of emotion in the relationship, we separated the analyses by emotions such that sensory reliance scores of the Angry condition or Happy condition were correlated with the recognition accuracy difference of face and voice. We found a significant correlation between sensory reliance and accuracy difference favoring the same modality in the Angry condition (Angry: *r* = 0.41, *p* = 0.004; Figure 5A), but not in the Happy condition (Happy: *r* = −0.23, *p* = 0.12; Figure 5B). This observation may lend support to the reliability-weighted model in Angry perception when the unity assumption is met. The reason this correlation was not observed in Happy emotion might be due to visual dominance in the Happy emotion as generally, happy faces were recognized more accurately than happy voices (Figure 5B). Comparing Figures 5A,B, it thus suggests an emotional effect in multisensory integration.

## Discussion

We investigated the criteria for multisensory integration through judgments of emotion from faces and voices at different intensities. We found that the more similar the audiovisual stimuli intensity, the more likely the unity assumption was, demonstrating the effects of cue disparity in a semantic context. There is an emotional effect in multisensory integration, with the Angry emotions observed to require more similar audiovisual intensities to form a “Unity” judgment as compared to the Happy emotions. The Angry emotion relied more on auditory stimuli whereas the Happy emotion relied more on visual stimuli. Lastly, we observed autistic traits to be unrelated to levels of unisensory reliance on one sensory modality for emotion perception in the present non-clinical population.

The major question we attempted to address in this study was the criterion of the semantic aspect of the unity assumption in emotion perception. In line with the maximum-likelihood model of Bayesian integration, we found that multisensory integration in semantic emotion perception follows a Gaussian distribution, with the presently identified criterion at around 2.5 points on a 7-point Likert scale in audiovisual emotional intensity difference. This is so as to directly reveal the multisensory integratory function, and to quantify the intensity differences in multisensory integration for semantic emotion perception. Interestingly, we also found the semantic criterion of the unity assumption to be more lenient for stronger faces than voices of the same emotion. Similar findings have been reported in studies investigating the temporal criterion of the unity assumption (Slutsky and Recanzone, 2001; Lewald and Guski, 2003; Chen and Vroomen, 2013). The sensory bias was explained as light typically reaching the perceiver earlier than sound since it travels faster, our brain therefore adjusting the time window for audiovisual integration to account for this difference. Our findings may suggest that facial emotion is accompanied with a weaker or less expressive voice emotion, or that facial emotion is given more weights on intensity rating than voice emotion. These possibilities may be further investigated in future studies.

Moreover, our findings also support that the unity assumption can modulate the audiovisual integration strategy used during audiovisual emotion perception. One tends to rely more on the information from visual inputs rather than auditory input during emotion perception when the unity assumption is met compared to when it was not. A similar finding was reported by Wallace et al. (2004), who showed that when audiovisual stimuli were judged as unified spatially, the spatial ventriloquist effect was completely biased toward visual information; whereas when they were judged as not unified, no bias, or even a negative bias was induced. One possible explanation is that once the unity assumption is met, the perceiver no longer cognitively calculates the average emotional intensity of the face-voice pair but instead perceives the face-voice pair as a whole, and this holistic percept is biased toward the dominating sensory inputs following the principles of multisensory integration, such as the reliability-weighted model (Ernst and Banks, 2002; Alais and Burr, 2004; Collignon et al., 2008; Chandrasekaran, 2017). In this study, visual input dominated when the unity assumption was met. This might have been due to the faces being more accurately recognized, and thus more reliable, than voices in the emotion identification task. Our findings in the angry perception condition support the reliability-weighted model. We found that those who could recognize angry faces more accurately than angry voices also tended to rely more on the faces than voices during audiovisual anger perception. However, this was not observed in the happy emotion, where only two of our subjects were more accurate in recognizing happy voices than faces. The lack of performance range in the happy emotion renders us unable to comment on the relationship between recognition accuracy and sensory reliance for happy perception at present.

Interestingly, our finding that angry emotion perception relies more on auditory stimuli while happy emotion perception relies more on visual stimuli suggests an emotion specific modality dominance effect. In previous studies examining the temporal and spatial criteria of the unity assumption, it was found that the audiovisual stimulus would be judged as coming from the same location, or at the same time if their temporal and spatial discrepancies were within −100 ms (sound first) to +300 ms (visual first) and at less than 15°, respectively, as indicated by the ventriloquism effect (Slutsky and Recanzone, 2001; Lewald and Guski, 2003; Chen and Vroomen, 2013). As above, sensory bias in the temporal criterion has been explained as being due to light generally reaching the perceiver earlier than sound, hence elongating time windows for adaptation in the brain (Slutsky and Recanzone, 2001). On the other hand, it was also suggested that our auditory sensory system dominates in the temporal domain and visual system dominates in the space domain (Choi et al., 2018). Therefore, the finding that angry emotion relies more on auditory stimuli may indicate a temporal dominance in the perception of anger.

A previous study by Collignon et al. (2008) reported audiovisual emotion perception to be generally visual dominant. Visual dominance, however, was not observed for both happy and angry emotions in our study. This may be due to differences in experimental design. For example, while the emotions of disgust and fear were used in Collignon et al.’s (2008) study, here we used anger and happiness. Further, the task implemented was different as well. While Collignon et al. (2008) asked subjects to identify the emotional *category* of audiovisual stimuli, we asked our subjects to rate the emotional *intensity* instead. Therefore, our instructions may have encouraged subjects to weigh the face and voice more equally during the task. With reference to the causal inference model, participants would have attributed both stimuli to a single, common cause, or C = 1 in this study, which should then produce an optimal estimate of stimulus intensity (Shams et al., 2010). Though an alternative thread to the causal inference model considers when a perceiver might perceive the two stimuli to be of separate causes, or *C* = 2, in which case intensity estimations are made based on separate percepts. Moreover, Collignon et al. (2008) conducted their study in Canada while the present study was conducted in Singapore, potentially suggesting a cultural effect on sensory reliance during multisensory emotion perception (Tanaka et al., 2010; Liu et al., 2015). Such cultural effects have been noted previously, with Japanese subjects observed to weight auditory emotional information more than the Dutch in audiovisual emotion perception (Tanaka et al., 2010). Similar cultural findings were replicated when contrasting Chinese and English speakers’ neural responses—the N400 signal more strongly interfering when judging emotionally incongruent faces than voices in English speakers compared to Chinese speakers (Liu et al., 2015). These cultural differences were suggested to result from differences in societal norms where East Asians may tend to mask expressions for indirectness in communication, in contrast to Western cultures (for more in-depth discussion, see Liu et al., 2015). Therefore, the type of emotion, judgment tasks and cultural difference may influence interpretations of findings across multisensory integration studies.

In this study, we also explored the influence of autistic traits on multisensory emotion perception in a non-clinical population. Surprisingly, we observed that autistic traits were not significantly correlated with unisensory processing ability when a single source was assumed. This appears contrary to findings of the Weak Central Coherence Theory (Happé and Frith, 2006). The Bayesian model of autistic perception postulates that the social/non-social perceptual deficits in autism result from deviation in the construction of top-down influences, i.e., priors, and/or the reductions in the use of such priors (Pellicano and Burr, 2012; Sevgi et al., 2020). Aligning with this model, a recent study suggested that autistic traits are associated with stricter temporal criteria for the unity assumption, one of the priors in multisensory perception (Körding et al., 2007; Shams and Beierholm, 2010; Chen and Spence, 2017), suggesting a deviated construction of the prior (Kawakami et al., 2020b). However, this association is not consistently observed in the literature (Kawakami et al., 2020a). Poole et al. (2017), for example, found no differences between adults with autism spectrum conditions and neurotypical individuals in temporal acuity. Similarly, de Boer-Schellekens et al. (2013) found no autistic influence in low-level audiovisual multisensory integration.

One potential explanation for this finding may be that the autistic influence occurs at a stage independent from top-down influences, specifically in the semantic aspect of audiovisual emotion perception of angry and happy faces (Chen and Spence, 2017). This impairment may be related to the social attention deficit in autism, which has been commonly reported in the literature. By reviewing results from these studies, Chita-Tegmark (2016) concluded that the autistic deficits in social attention is likely the result of the difficulty faced in monitoring large number of social stimuli, e.g., humans, or social interactions. This would lead us to expect autistic individuals to be unable to successfully attend to social information from both visual and auditory channels simultaneously. The observed results may potentially be attributed to the nature of study stimuli, involving recognition of emotional faces and voices. The exact relationship between autistic traits and emotional recognition in faces remains inconclusive. For example, in a clinical sample, Cook et al. (2013) found autism and autistic spectrum conditions to be unrelated with facial emotion perception ability, irrespective of gender, IQ, and age. Further, Ola and Gullon-Scott (2020) also noted impaired facial emotion recognition to be associated with alexithymia, but not with autistic traits. Interestingly, despite the well-reported impairments of autistic traits in multisensory processing, this was not observed in the instance of audiovisual emotion integration. Especially as the literature remains sparse in this regard, further understanding may elucidate the complex link between facial and auditory emotion perception in multisensory integration for autistic individuals and the general population.

The current study presents a few limitations. First, here we referred to the unity assumption as a dichotomous process, though recent research suggested a continuous nature of processing (Chen and Spence, 2017). Next, as mentioned above, we did not presently account for the segregated percept (*C* = 2) branch, and modeling both branches may gain a full spectrum of intensity percepts. We expect these to be addressed in future studies, which may also investigate the exact mechanisms of multisensory integration for autistic individuals (by disrupted holistic perception, attenuated priors, reduced weights, social attention deficits, or other possibilities) in a semantic emotion context.

## Data Availability Statement

The raw data supporting the conclusions of this article will be made available by the authors, without undue reservation.

## Ethics Statement

The studies involving human participants were reviewed and approved by Institutional Review Board (IRB) at Nanyang Technological University, Singapore. The participants provided their written informed consent to participate in this study.

## Author Contributions

KS and HX designed the experiment. KS created the stimuli and collected and analyzed the data in the first draft of the manuscript. AS collected and analyzed the data in the following drafts. KS wrote the first draft of the manuscript with the other authors responsible for manuscript revisions and comments. All authors contributed to the article and approved the submitted version.

## Conflict of Interest

The authors declare that the research was conducted in the absence of any commercial or financial relationships that could be construed as a potential conflict of interest.

## Publisher’s Note

All claims expressed in this article are solely those of the authors and do not necessarily represent those of their affiliated organizations, or those of the publisher, the editors and the reviewers. Any product that may be evaluated in this article, or claim that may be made by its manufacturer, is not guaranteed or endorsed by the publisher.
